# Remote Interviewer Training for COVID-19 Data Collection: Challenges and Lessons Learned From 3 Countries in Sub-Saharan Africa

**DOI:** 10.9745/GHSP-D-20-00468

**Published:** 2021-03-31

**Authors:** Shani Turke, Sarah Nehrling, Samuel Olanipekun Adebayo, Pierre Akilimali, Ivan Idiodi, Anthony Mwangi, Elizabeth Larson, Caroline Moreau, Philip Anglewicz

**Affiliations:** a Bill & Melinda Gates Institute for Population and Reproductive Health, Department of Population, Family, and Reproductive Health, Johns Hopkins Bloomberg School of Public Health, Baltimore, MD, USA.; b Garabam Consulting, Cambridge, MA, USA.; c Centre for Research, Evaluation Resources and Development, Abuja, Nigeria.; d Kinshasa School of Public Health, Kinshasa, Democratic Republic of the Congo.; e International Centre for Reproductive Health, Mombasa, Kenya.; f Department of Population, Family, and Reproductive Health, Johns Hopkins Bloomberg School of Public Health, Baltimore, MD, USA.; g Soins et santé. Center for Research in Epidemiology and Population Health, INSERM 1018 Villejuif, France.

## Abstract

There is an urgent need for data to inform coronavirus disease (COVID-19) pandemic response efforts. At the same time, the pandemic has created challenges for data collection, one of which is interviewer training in the context of social distancing. In sub-Saharan Africa, in-person interviewer training and face-to-face data collection remain the norm, requiring researchers to think creatively about transitioning to remote settings to allow for safer data collection that respects government guidelines. Performance Monitoring for Action (PMA, formerly PMA2020) has collected both cross-sectional and longitudinal data on key reproductive health measures in Africa and Asia since 2013. Relying on partnerships with in-country research institutes and cadres of female interviewers recruited from sampled communities, the project was well-positioned to transition to collecting data on COVID-19 from the onset of the pandemic. This article presents PMA's development of a remote training system for COVID-19 surveys in the Democratic Republic of the Congo, Kenya, and Nigeria, including challenges faced and lessons learned. We demonstrate that remote interviewer training can be a viable approach when data are critically needed and in-person learning is not possible. We also argue against systematic replacement of in-person trainings with remote learning, instead recommending consideration of local context and a project's individual circumstances when contemplating a transition to remote interviewer training.

## Abstract (precis)

Remote training of interviewers in low-resource settings can be an effective approach during the COVID-19 pandemic when data are critically needed and in-person learning is not possible. We demonstrate that remote interviewer training is possible when interviewers: have at least an intermittent Internet connection, have select physical materials available, and are experienced and part of a cohesive team.

## Teaser

**Key Findings:**

- Remote interviewer training for large-scale surveys can be an effective replacement to in-person learning in low-resource contexts when data are urgently needed and in-person learning is impossible.
- An intermittent Internet connection, distribution of physical materials to interviewers, and interviewer experience and social cohesion are critical to the success of remote training.

**Key Implications:**

- Training designers need to balance streamlining design choices for efficiency and the importance of contextual adaptation to improve training quality.
- In-person learning still conveys significant advantages over remote learning. Training designers should evaluate local context and a project's unique circumstances before proceeding with remote training.

Résumé en français à la fin de l'article.

## INTRODUCTION

Since the coronavirus disease (COVID-19) pandemic began, researchers have repurposed ongoing survey platforms to provide timely data and inform COVID-19 response efforts.^1^^–^^8^ Transitioning toward COVID-19 research is particularly important for countries in sub-Saharan Africa, where the limitations of disease surveillance systems were well known even before the pandemic.^9^^,^^10^ Existing survey platforms can offer critical support to COVID-19 monitoring efforts; however, rapid repurposing of survey infrastructure is not without challenges.

In much of sub-Saharan Africa, in-person interviewer training and face-to-face data collection remain the norm for population-based surveys, largely due to inconsistent Internet connectivity and barriers to technological literacy.^11^^,^^12^ However, during the COVID-19 pandemic, in-person activities may violate government restrictions and put respondents at risk of infection. As a result, some surveys have shifted to abbreviated remote trainings or have forgone formal interviewer training altogether.^2^^–^^4^^,^^6^^,^^13^^,^^14^ Comprehensive interviewer training impacts data quality, so it is important to consider remote training options under the current circumstances.^15^^–^^20^ To date, little has been published on remote interviewer training in low-resource contexts, and what does exist lacks detail or is largely anecdotal.^2^^–^^4^^,^^6^^,^^13^ Comprehensive documentation is necessary to better understand when remote training may be feasible and to establish best practices for remote learning in these settings.

Comprehensive interviewer training impacts data quality, so it's important to consider remote training options during the COVID-19 pandemic.

## PERFORMANCE MONITORING FOR ACTION

Performance Monitoring for Action (PMA, formerly PMA2020) has collected rapid-turnaround data on family planning and other reproductive health indicators across Africa and Asia since 2013. Universities and research institutes lead implementation in each country (referred to as “country teams” in this article), recruiting female interviewers from sampled communities to conduct mobile phone-based population and facility surveys annually. Data are representative at the national or subnational level and provide timely information to policy makers on key indicators in sexual and reproductive health. The Johns Hopkins Bloomberg School of Public Health (JHSPH) and Jhpiego provide technical and coordination support. Beginning in late 2019, PMA transitioned to a panel design, with annual, in-person follow-up surveys planned over 3 years.

Preparing for PMA's baseline survey begins with a 2-week in-person training for the country team project staff and field supervisors, followed by a 2-week training of interviewers. Before each subsequent survey round, teams hold shorter refresher trainings, typically between 3 and 5 days, to review challenges from the previous round and train on new survey topics. Facilitators employ a variety of formats, including lecture, small group activities, individual assessments, and field exercises.

In late 2019 and early 2020, teams in 4 of the 8 PMA countries—Burkina Faso, the Democratic Republic of the Congo (DRC), Kenya, and Nigeria—collected baseline survey data in-person, obtaining consent and phone numbers from women willing to participate in follow-up surveys. These teams also had previous experience with conducting remote surveys over the phone as part of PMA Agile, a separate project within the PMA platform.^21^

Given this background, PMA was well-poised to collect COVID-19 data remotely through phone interviews and attempt remote interviewer trainings. In March 2020, PMA developed a COVID-19 survey and began preparing for remote training. In this article, we present PMA's remote interviewer training approach implemented in 3 of the 4 countries: the Democratic Republic of the Congo (DRC), Kenya, and Nigeria. We also describe key challenges we faced and provide recommendations for others considering remote interviewer training.

We describe here the PMA COVID-19 survey, the key features of PMA's approach to remote interviewer training, an interviewer's typical training experience, and country-specific adaptations to the training system.

### The PMA COVID-19 Survey

PMA's COVID-19 survey consisted of a short phone-based questionnaire administered to women of reproductive age (15–49 years) who consented to follow-up at baseline. The survey contained 6 sections: COVID-19 awareness and information sources; perception of personal infection risk; knowledge of COVID-19 symptoms, transmission, and prevention; social consequences of COVID-19-related restrictions; and the impact of COVID-19 on accessing health services as well as reproductive health outcomes. Data are representative of Kinshasa province in the DRC, nationally representative in Kenya, and representative of Kano and Lagos States in Nigeria. PMA is led by the Kinshasa School of Public Health (KSPH) in the DRC; the International Centre for Reproductive Health (ICRH) in Kenya; the Centre for Research, Evaluation Resources, and Development (CRERD) in Nigeria; and the *Institut Supérieur des Sciences de la Population* in Burkina Faso.

### PMA's Remote Training System

As we began planning for PMA's COVID-19 survey, we considered our options for interviewer training. Country teams weighed the feasibility of interviewers participating in training from their homes against the practicality and safety of in-person trainings during the pandemic. We recognized the health risks to in-person trainings, while also acknowledging the risk of poor data quality if we chose to conduct minimal or no training at all. We knew quality issues would extend the cleaning phase for urgently needed data, thus limiting the survey's overall utility. Ultimately, teams in the DRC, Kenya, and Nigeria elected to conduct training remotely, with technical support from JHSPH staff and an independent learning consultant.

Country teams weighed the feasibility of remote interviewer training from homes against the practicality and safety of in-person trainings during the pandemic.

As training approached in Burkina Faso, new COVID-19 cases waned, and government restrictions relaxed. As such, the *Institut Supérieur des Sciences de la Population* proceeded with in-person, socially distanced training following PMA's standard training procedures. We do not present information here on this latter approach.

Recognizing that interviewers had limited familiarity with online learning, our goal was to develop a training system that mimicked the in-person experience. This included sharing content through video lectures, reinforcement through small group activities, evaluation through electronic assessments, and active monitoring via one-on-one phone calls between facilitators and interviewers. Interviewers accessed all training materials from PMA smartphones also used for data collection. To reduce interviewers' learning burden, we relied on platforms with which they were already familiar, namely WhatsApp, Google Drive, Open Data Kit, and YouTube.

Each country team had its own distinct training system, based around 3 types of WhatsApp groups, named, “Info,” “My Group,” and “Q&A,” for information-sharing, small group work, and asking questions, respectively (Figure 1). The WhatsApp “Info” group served as the “training room,” or the central location where interviewers could follow along with the training agenda and access training content. It was designed as a unidirectional group, where only the lead trainer and facilitators could post. This made the group the centralized place for information-sharing, whereas the “Q&A” and “MyGroup” WhatsApp groups were designed for bidirectional exchange and interaction between interviewers and facilitators.

**FIGURE:**
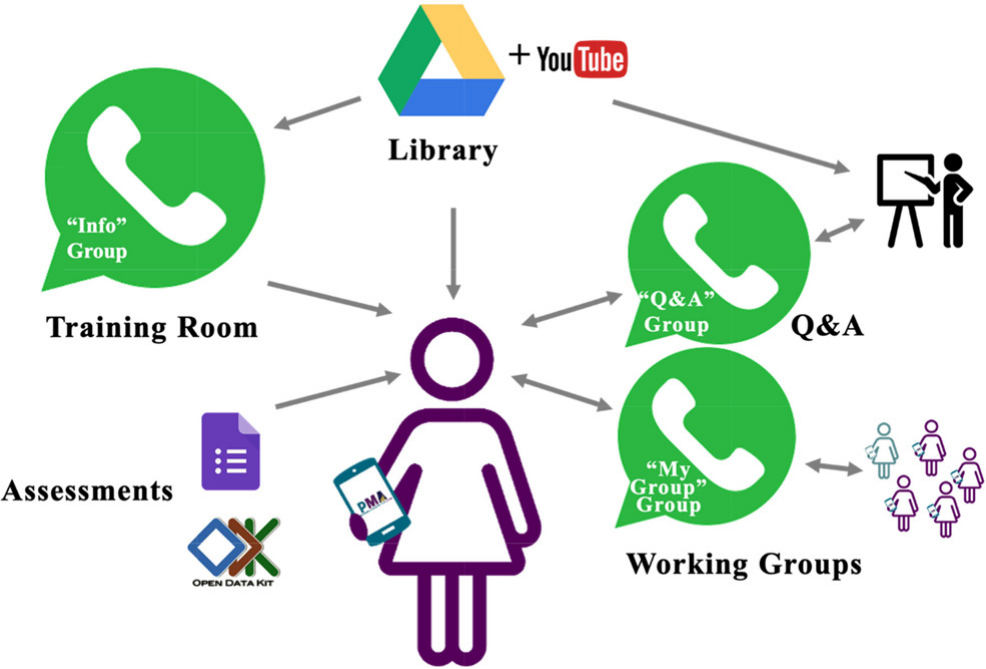
The Remote Interviewer Training System for the Performance Monitoring for Action COVID-19 Survey, March 2020

At the start of each training day, lead facilitators began with a virtual roll call in the “Info” group and posted an image of the day's agenda. They then shared links to the training content for each session, which video lectures previously recorded and uploaded to the PMA YouTube channel, as well as activity instructions, and links to individual assessments. They posted training content to the “Info” group in the order given in the agenda, with time between each post for interviewers to watch the videos or complete the activity. If interviewers had questions, they posted them to the “Q&A” WhatsApp group. Small group activities were completed in their assigned “My Groups” WhatsApp group. Interviewers completed assessments using Open Data Kit or GoogleForms that facilitators then graded. The lead trainer moderated the “Q&A” group and coordinated with facilitators moderating the “MyGroups” and grading assessments to post any information that was relevant to all interviewers to the “Info” group.

Knowing that Internet connectivity would pose a challenge, country teams downloaded backup copies of training content to the interviewers' smartphones via the Google Drive app before distributing the smartphones to interviewers. We also did not include any live sessions in the original training design, though some country teams chose to add group video calls as their Internet connection permitted. We distributed printed training manuals to ensure that interviewers had a reference text while watching videos and completing activities, and as a final backup option if all technology failed. Given the pressing need for these data, we accelerated training material development by having JHSPH staff and the independent consultant create ready-to-implement training lectures and activities, with options for country-specific adaptation. We designed training lectures to be no longer than 20 minutes and made them as interactive and participatory as possible, given the virtual setting. Remote trainings in all 3 countries took place over 3 consecutive days between May and July 2020. Details of the training experience from an interviewer's perspective are shown in Box.

BOX **An Interviewer's Typical Experience with Remote Training for the Performance Monitoring for Action COVID-19 Survey**

1. **Receive physical materials:** Included a printed training manual and configured smartphone, with copies of training materials synced locally for offline viewing on Google Drive. For ease of access, we added shortcuts of the Google Drive training folder and other relevant applications to the home screen of each phone.
2. **Orient to remote training system:** Facilitators shared the day's agenda in the unidirectional WhatsApp “Info” group, pushing content for each session in chronological order throughout the training day. Training tools had unique identification numbers corresponding to the day and sequence of the session. Interviewers accessed tools from links shared by WhatsApp group or by searching by identification numbers in Google Drive.
3. **Learn content:** Interviewers watched videos, pausing for pop quizzes and reminders to review their training manual.
4. **Apply content and evaluate understanding:** Facilitators shared pre-designed activities in the WhatsApp “Info” group, such as practicing survey introduction with a member of the interviewer's household or another interviewer over the phone. Interviewers completed activities and took daily Google Forms or Open Data Kit quizzes and received automatic grading and individualized feedback.
5. **Ask questions and receive support:** Interviewers asked questions and received responses via multidirectional exchange on the WhatsApp “Q&A” group. They communicated in smaller WhatsApp groups called “MyGroup [#]” for direct support from facilitators. Field supervisors tracked progress and comprehension via a shared spreadsheet that centralized data on homework completion and quiz scores. Supervisors followed up with interviewers by phone as needed.

Because Internet connectivity would pose a challenge, country teams distributed print manuals, adapted training to include recorded, not live, sessions, and designed training lectures to be 20 minutes or less.

### Country-Specific Adaptations

While adhering to the general training system described above, each country team adapted aspects of this remote approach to make it more effective in their context. In Kenya, ICRH recruited all available interviewers who participated in the baseline survey (n=282). They sent preloaded smartphones and training manuals via courier service to reduce infection risk. Given the large team, ICRH developed a cascading training approach, starting with a remote training of trainers (TOT) that included participation from experienced PMA Agile interviewers and field supervisors. The TOT followed the general remote training model as described. For interviewer training, each county organized separate trainings with its own WhatsApp groups “Info,” “MyGroup,” and “Q&A.” Field supervisors who had participated in the TOT moderated the county-specific groups and sent questions they could not answer to the TOT “Q&A” group. They relayed responses back to interviewers via the county groups, thus standardizing training content for all interviewers.

In the DRC, KSPH recruited a subset of 25 interviewers who had participated in the baseline survey and/or PMA Agile. To translate the COVID-19 questionnaire into Lingala, a language not widely written, the team invited interviewers to participate in a pretraining competition. Interviewers submitted audio files of their questionnaire translations in Lingala. The central team reviewed submissions and sent the most accurate and specific translations to all interviewers as the standard translation for each question.

In Nigeria, CRERD recruited a subset of 10 interviewers in Kano State and 21 in Lagos State, all of whom participated in baseline data collection. They also recruited 2 supervisors from Kano State and 4 from Lagos State to manage teams of interviewers during training and data collection. Field coordinators for each state distributed preloaded smartphones and training manuals, rather than asking interviewers to come to a central location and risk infection. The team translated the COVID-19 questionnaire into Pidgin, Hausa, and Yoruba. Daily, live group video calls enabled interviewers to socialize and discuss training content further. CRERD continued using the WhatsApp “Q&A” group during data collection to reinforce learning and enhance general communication.

## CHALLENGES TO REMOTE TRAINING

In the implementation of our remote training system, we encountered 3 central challenges. We describe these challenges and our attempts to address these issues in real-time.

### How Do We Rapidly Prepare Remote Training Facilitators in Each Country?

Country teams already had extensive experience leading in-person interviewer trainings, but facilitating a virtual training required additional skills. Given the urgent need for the data, we knew we had limited time to support this learning. Acknowledging the time constraints, how could we help build facilitators' confidence with virtual training?

In an attempt to reduce the learning burden on facilitators from the beginning, we designed a training system that leveraged participants' existing technological knowledge, using applications like WhatsApp that most interviewers and facilitators already used. In Kenya, the ICRH team built in additional experience by having interviewers who had conducted remote surveys under PMA Agile participate in the TOT and then serve as lead facilitators for the remote, county-level trainings. In the DRC, where the KSPH team was particularly concerned about interviewers' comfort using WhatsApp, facilitators pretested a prototype of the remote training system even before deciding to adopt a remote approach. This increased facilitators' confidence in using the training system while improving the overall design.

To try to reduce the facilitators' learning burden, we designed a training system that leveraged participants' existing technological knowledge by using apps that most interviewers and facilitators already used.

Still, we acknowledge that the preparation process was not perfect, and we omitted steps that could have helped country teams better prepare their trainings. Country teams expressed post-training that they would have benefited from an introductory webinar presenting the training system in its entirety and how we envisioned it being used. Due to time constraints, we instead provided this information in piecemeal, usually over email. This created confusion about the intended order of training sessions, which training sessions we envisioned country teams adapting or creating, and duplication of efforts between JHSPH staff and country teams. We also did not provide sufficient training on new tools introduced as part of the remote training approach. For example, we introduced Google Forms for virtual assessments but did not offer guidance on how to use it. As a result, some country teams did not know about key features that made it ideal for remote learning, such as automatic grading and immediate question-by-question feedback, until later in the training.

### How Do We Ensure That Interviewers Are Able to Fully Participate in the Training?

We were uncertain of the extent to which interviewers would be able to participate in training from their homes. We were particularly worried about Internet connectivity, which ranged from generally stable with sufficient bandwidth to frequent outages and low bandwidth. We were also concerned about interviewer availability, given other household obligations for which women are often responsible.

In response to Internet connectivity concerns, we invested early in testing the technology necessary for remote training, while also preparing several backup options for when the Internet inevitably failed. In the overarching training system, country teams downloaded offline copies of all training materials to the smartphones ahead of distribution. In Nigeria, the team conducted a 2-stage verification by ensuring that videos were playing offline before and after delivering phones to interviewers. Interviewers could also view videos via PMA's YouTube channel, which automatically adjusts streaming quality based on available bandwidth. Hard copies of the training manuals served both as a reference text and an additional low-tech option for learning.

**FIGURE 2:**
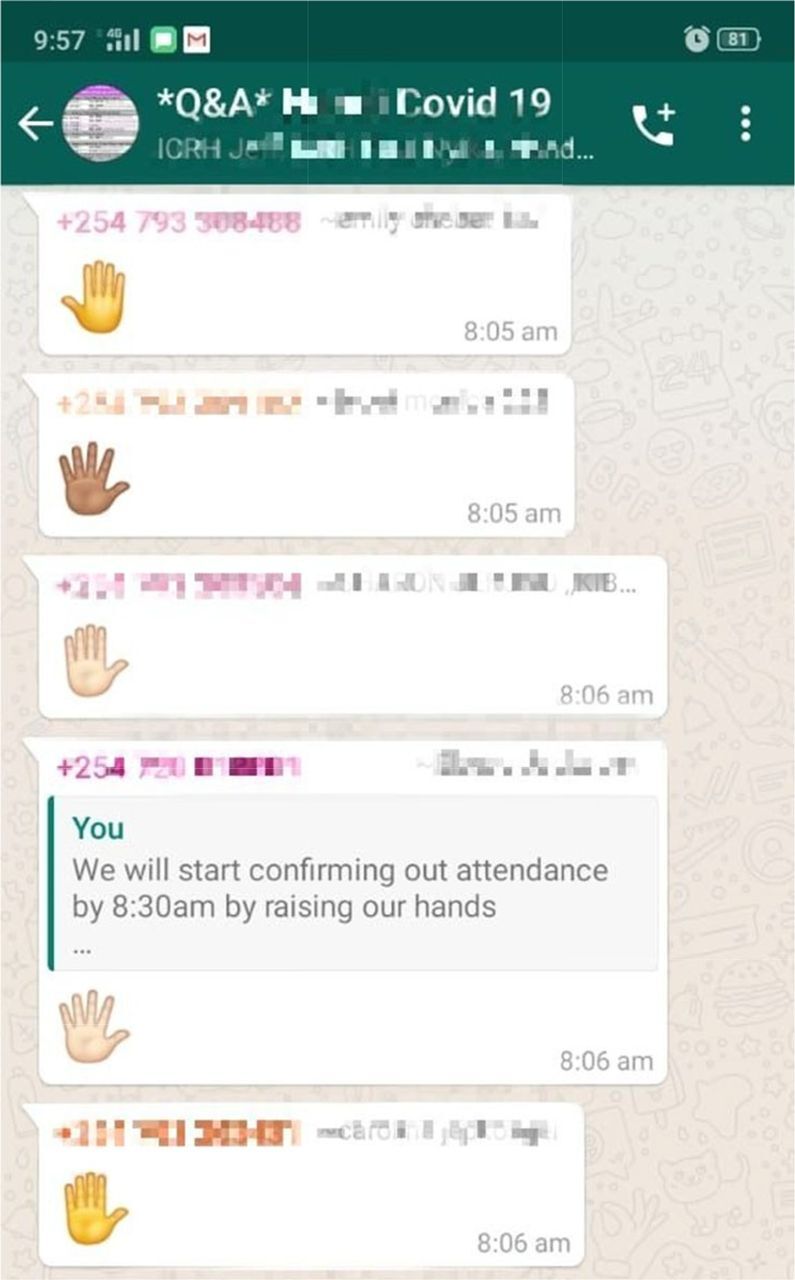
Screenshot of the Virtual RollCall Implemented by the International Centre for Reproductive Health in Kenya to Assess if Interviewers Were Present and Following the Remote Training.

Recognizing that competing household priorities was also not an insignificant challenge for a group of female interviewers, all teams adopted a strategy developed by ICRH. This approach allowed interviewers to go through a day's training at their own pace, while requiring everyone to finish all sessions for the day by a designated time. Interviewers thus had the flexibility to fit training around household responsibilities, while still benefitting from synchronous learning. ICRH also implemented a roll call to start each training day, where a facilitator asked interviewers to virtually raise their hand by sending a hand emoji to the WhatsApp “Q&A” group, acknowledging they were online and ready to start training (Figure 2).

### How Do We Ensure Interviewers Are Actually Learning?

Beyond basic participation, we saw evaluation of interviewer performance as another central challenge. How could we ensure that interviewers understood and retained the material they accessed remotely?

As part of the training system, we created quizzes in Google Forms and Open Data Kit and tracked performance with a shared spreadsheet. In addition, country teams developed ways to routinely gauge interviewer comprehension. The CRERD team used the small-group format of the WhatsApp “MyGroups” to delve deeper into confusing topics. Live evening debriefs over video call covered lingering issues that could not be resolved in these small groups. The review meetings served both to clarify content for interviewers and also to verify comprehension. For example, when interviewers raised questions, the facilitator randomly chose another interviewer to respond before providing further clarity.

Country teams developed different ways to gauge interviewers' comprehension including small group discussions via Whatsapp and recorded, live debriefs via video call.

In the DRC, the KSPH team announced at the beginning of training that 2 interviewers would be selected at the end of each training day to provide a daily summary the following morning. This created an incentive for interviewers to complete the day's training on time, clarify any confusing points, and be prepared if they were selected to report out the next day. Finally, all teams acknowledged that despite the numerous options for tracking learning virtually, the best way to support struggling interviewers was often still to call them and provide one-on-one support.

## LESSONS LEARNED

We share our approach and the challenges we faced in implementing remote interviewer trainings for a COVID-19 survey in the DRC, Kenya, and Nigeria. We hope this information will assist other groups planning data collection activities during the pandemic. To this effect, we discuss below 2 overarching lessons learned from the experience.

### Acknowledge Trade-offs Between Country Ownership and Timelines

Given that the data were urgently needed, we struggled with the tension between (1) fostering local ownership over training design and (2) meeting timelines for data collection. Centralized decision making by JHSPH staff in the name of efficiency led to some confusion that impacted training quality. An incomplete understanding of why we chose certain training tools made it difficult for country teams to decide which approaches to implement and how best to adapt them to their contexts. When there was time for country adaptation, it was also evident that teams' additions improved interviewer participation and knowledge retention. Daily virtual roll calls, random interviewer selection to provide daily summaries, and live video calls all enhanced the overall training experience by ensuring participation, assessing comprehension, and building a sense of in- and cross-country comradery. Although we have little information on remote interviewer trainings, we know from the literature on interviewer training more generally that these factors are associated with better interviewer performance and improved data quality.^16^^–^^18^

Given this, we wished we had invested more time upfront toward greater inclusivity in the training design process. A core tenet of PMA's design centers on promoting country team autonomy in survey implementation over time. We approached remote training development with this goal in mind, and yet still struggled. Ideally, JHSPH staff and the learning consultant would have cocreated the training system with country team facilitators. We would have taken more time to coach country teams on the proposed approach and the logic behind the design choices, and we would have created more space for feedback and new design options by country teams.

We recognize that this polarity is not unique to PMA nor is it unique to remote learning. However, circumstances like the COVID-19 pandemic that necessitate remote learning in low-resource contexts will likely only exacerbate this common tension further. As such, we recommend that others facing similar constraints model the importance of a minimal viable product for content creation.^22^ The minimal viable product concept originated in business, as a way for technology startups to bring new technology to market faster by introducing a product once it met minimal useability and functionality standards. This process accelerated users' access to a functional product while allowing designers to collect user data to make iterative product improvements. Promoting the concept of a minimal viable product in the context of remote training design is helpful because it demonstrates that imperfect, unpolished materials can be created in less time, while still being effective for learning. We also suggest that training system designers invest their time engaging with country teams earlier in the planning, specifically by organizing a facilitator orientation session to introduce the system and get feedback before getting into the details of content creation. When data are urgently needed, an honest and collaborative assessment of the trade-off between local ownership and efficiency is even more critical, along with creative solutions to strike a balance between the 2 sides of the polarity.

Promoting the concept of a minimal viable product in the context of remote training design is helpful because it demonstrates that imperfect, unpolished materials can be created in less time, while still being effective for learning.

### Thoughtful Consideration Over a Uniform Approach

We summarize our identified advantages and limitations of remote compared to in-person trainings, along with factors to consider when deciding on an approach (Table). Remote training conveys some advantages over in-person training under specific circumstances. Most fundamentally, it allows for some level of interviewer training with minimal added infection risk. Secondary benefits of remote learning among our global team included increased confidence in using the tools of remote learning and easier cross-country and cross-language collaboration to improve the overall training design. Having training content recorded also meant that interviewers could watch training lectures multiple times to increase retention.

**TABLE.** Advantages and Limitations of Implementing Remote Interviewer Trainings and Factors to Consider When Deciding on Approach

Remote Training Advantages	Remote Training Limitations
Allows some interviewer training safely during a global pandemic Builds confidence, skills, and familiarity with remote training, to leverage future remote learning if necessary Enables cross-country and cross-language knowledge sharing to create standardized materials Enables participants to watch prerecorded content multiple times and on their own schedule	Requires preparing multiple back up options in anticipation of Internet connectivity or technology issues Necessitates additional training in remote facilitation for training facilitators Limits opportunities for organic learning from discussion or practical application Demands significant human resources to design, develop, and deploy the training systemRequires group tolerance for the risks and potential delays
**Ideal Circumstances for Remote Training**	**Circumstances That Do Not Favor Remote Training**
Access to at least an intermittent Internet connection Ability to distribute select materials to interviewers, such as a training manual and smartphone, to engage in remote learning An established and cohesive field team, enabling an environment conducive to attempting new ways of learning Experienced interviewers, with sufficient background knowledge on protocols and survey content to minimize training time Simple survey content that builds on interviewers' previous experiences Familiarity across the study team with at least a few common applications that could be used for remote learning	Internet connection is unavailable or unreliable for large portions of training time Printed materials and common familiar applications cannot be ensured Field team is new, or trust and familiarity are still being developed across a team Training content is lengthy or significantly complex In-person practical application is essential to learning

Nonetheless, it is important to acknowledge the limitations of remote trainings and that in-person learning has significant pedagogical advantages. These include easier observation of learner engagement, greater flexibility to address learners' needs throughout training, more opportunities for organic discussion and practical exercises, and greater socialized learning alongside other learners.^15^^–^^17^^,^^23^ Other groups implementing COVID-19 surveys with remote interviewer training have also noted disadvantages to self-directed learning in the home environment compared to in-person trainings.^6^^,^^14^ Finally, others considering implementing a remote approach should be aware of the extensive time investments needed to develop an effective virtual training, especially if aiming for standardization across geographies.

In terms of factors that enable successful remote learning, our ability to effectively mitigate many of the challenges above was largely due to PMA's structure and history as a project. We have invested considerable time and resources into interviewer training and fostering an environment of collaboration across the project. Most interviewers remain with the project over many survey rounds, meaning those who participated in the COVID-19 survey had substantial existing knowledge of PMA survey protocols, research ethics, and phone-based data collection. Country teams have also made significant efforts to develop a strong sense of community among field teams, which undoubtedly contributed to interviewer confidence in attempting a new way of learning. Guidelines on conducting remote interviewer trainings from the World Bank, Innovations for Poverty Action, and Abdul Latif Jameel Poverty Action Lab likewise note the importance of having an experienced and cohesive field team as well as familiar, easily accessible technology.^4^^,^^6^^,^^13^^,^^14^ Though critical to PMA's success with remote learning, these factors may not be present or feasible for other large-scale surveys.

For these reasons, we do not advocate for systematically replacing in-person trainings with remote learning. Instead, thoughtful consideration of the specific circumstances and the project's available resources are needed when deciding on the feasibility of conducting interviewer trainings remotely. Indeed, JHSPH staff and principal investigators in each country discussed the above considerations early in the planning process, and colleagues in Burkina Faso ultimately decided to hold an in-person training. The team explained that Internet connectivity was not consistent or reliable enough even in the capital of Ouagadougou for remote training to be a viable option. In their case, an in-person training with strict safety and infection protocol measures was feasible and still safe. Everyone who attended training was given masks and hand sanitizer and instructed to remain appropriately spaced. Instead of large plenaries, the team held training in small groups and worked outdoors whenever possible. Survey teams implementing in similar settings during the COVID-19 pandemic may want to consider such an approach. Under more typical circumstances, teams implementing a survey for the first time or with entirely new interviewers should also think about extending in-person trainings with remote learning, rather than replacing them entirely.

## CONCLUSION

This article demonstrates that when data are urgently needed and in-person interviewer trainings are not possible, a well-designed remote training can be an effective replacement in low-resource contexts in sub-Saharan Africa. Development of our remote training approach was not without challenges, including difficulties rapidly preparing for remote facilitation, and ensuring interviewer participation and comprehension from home. Critical factors to the success of our design include an experienced and cohesive team of facilitators and interviewers, the ability to rapidly distribute select physical training materials, and interviewer access to an intermittent Internet connection at a minimum. Although we do not advocate for systematic replacement of in-person trainings with remote learning, demonstrating that a remote approach is possible in these settings is an important step toward ensuring the availability of high-quality data during the COVID-19 pandemic.

## Translation

**En Français**

**Formation à distance des enquêteurs pour la collecte de données sur la COVID-19: Défis et leçons tirées de 3 pays d'Afrique subsaharienne**

Messages clés: La formation à distance des enquêteurs dans des environnements à faibles ressources peut être une approche efficace pendant la pandémie de la COVID-19 lorsque les données sont absolument nécessaires et que l'apprentissage en personne n'est pas possible. Nous démontrons que la formation à distance des enquêteurs est possible lorsque les enquêteurs: disposent au moins d'une connexion Internet intermittente, disposent de supports matériels spécifiques, sont expérimentés et font partie d'une équipe soudée.

**ABSTRACT**

Il y a un besoin urgent de données pour informer les efforts de réponse à la pandémie de coronavirus (COVID-19). En même temps, la pandémie a créé des défis pour la collecte de données, dont l'un est la formation des enquêteurs dans le contexte de la distanciation sociale. En Afrique subsaharienne, la formation des enquêteurs en personne et la collecte de données en face à face restent la norme, ce qui oblige les chercheurs à réfléchir de manière créative à la transition vers des cadres de travail à distance afin de permettre une collecte de données plus sûre et respectant les directives gouvernementales. Depuis 2013, Performance Monitoring for Action (PMA, anciennement PMA2020) a permis de recueillir des données transversales et longitudinales sur les principaux indicateurs de la santé reproductive en Afrique et en Asie. S'appuyant sur des partenariats avec des instituts de recherche nationaux et des équipes d'enquêtrices recrutées dans les communautés échantillonnées, le projet était bien positionné dès le début de la pandémie pour passer à la collecte de données sur la COVID-19. Cet article présente le développement par PMA d'un système de formation à distance pour les enquêtes COVID-19 en République Démocratique du Congo, au Kenya et au Nigeria, y compris les défis rencontrés et les leçons apprises. Nous démontrons que la formation des enquêteurs à distance peut être une approche viable lorsque les données sont absolument nécessaires et que l'apprentissage en personne n'est pas possible. Nous argumentons également contre le remplacement systématique des formations en personne par l'apprentissage à distance, et recommandons plutôt de prendre en compte le contexte local et les circonstances individuelles d'un projet lorsque l'on envisage une transition vers la formation à distance des enquêteurs

**Peer Reviewed**
